# Functional profiling of stage-specific proteome and translational transition across human pre-implantation embryo development at a single-cell resolution

**DOI:** 10.1038/s41421-022-00491-2

**Published:** 2023-01-24

**Authors:** Yujiao Dang, Liu Zhu, Peng Yuan, Qiang Liu, Qianying Guo, Xi Chen, Shuaixin Gao, Xiao Liu, Shushen Ji, Yifeng Yuan, Ying Lian, Rong Li, Liying Yan, Catherine C. L. Wong, Jie Qiao

**Affiliations:** 1 Center for Reproductive Medicine, Department of Obstetrics and Gynecology, Center for Precision Medicine Multi-Omics Research, Third Hospital, Peking University, Beijing, China; 2 National Clinical Research Center for Obstetrics and Gynecology, Peking University Third Hospital, Beijing, China; 3 Key Laboratory of Assisted Reproduction (Peking University), Ministry of Education, Beijing, China; 4 Beijing Key Laboratory of Reproductive Endocrinology and Assisted Reproductive Technology, Beijing, China; 5 Research Units of Comprehensive Diagnosis and Treatment of Oocyte Maturation Arrest, Beijing, China; 6 Peking-Tsinghua Center for Life Sciences, Beijing, China; 7 Department of Medical Research Center, State Key Laboratory of Complex Severe and Rare Diseases, Peking Union Medical College Hospital, Chinese Academy of Medical Science & Peking Union Medical College, Beijing, China

**Keywords:** Proteomics, Developmental biology

Dear Editor,

Pre-implantation development is the first step in giving rise to a new life. The landscape of epigenomic^1,2^ and transcriptomic^3,4^ regulation during human pre-implantation development has been mapped by applying single-cell sequencing. In contrast, investigations of the embryonic proteome are severely limited due to the insufficiency of precious human embryonic samples for traditional mass spectrometry (MS). The technology for the single-cell proteomics (SCP) has lagged behind single-cell sequencing due to protein loss during pretreatment and impossible amplification of peptides in MS. During the stages of pre-implantation development, the size of a single cell reduces from ~120 µm of an oocyte to ~15 µm of a blastomere in a blastocyst^4,5^. The quantity of corresponding proteins decreases from 100 ng to ~100 pg^6^. This creates great technical challenges for proteomic investigation on human embryos.

To break through the technological bottleneck, we have applied the state-of-the-art ultrahigh-sensitivity MS technology and nanoliter-scale oil-air-droplet (OAD) chips^7^ to realize SCP and were able to identify thousands of proteins in a single cell during human pre-implantation development. We collected a total of 58 samples of single oocytes or blastomeres for proteome analysis at seven crucial stages during human early embryo development (Fig. 1a; Supplementary Table S1). Based on proteomic profile, we identified approximately three thousand proteins in a single oocyte (Fig. 1b). Along embryonic development, due to rapid cell division and decreased cell size, the number of encoded proteins was reduced to approximately one thousand (1141) in single cells from blastocysts. The average correlation coefficient was ~0.7 among duplicates at each stage, indicating good reproducibility (Supplementary Fig. S1a, b). Principal component analysis (PCA) (Fig. 1c) and unsupervised hierarchical clustering (Supplementary Fig. S1c) showed that samples fitted well to the corresponding stages of human embryo development, with the exception of three cells from morula-stage embryos which clustered with neighboring 8-cell blastomeres, indicating relatively high similarity of proteomic profiles between 8-cell embryos and morulae.

**Fig. 1:**
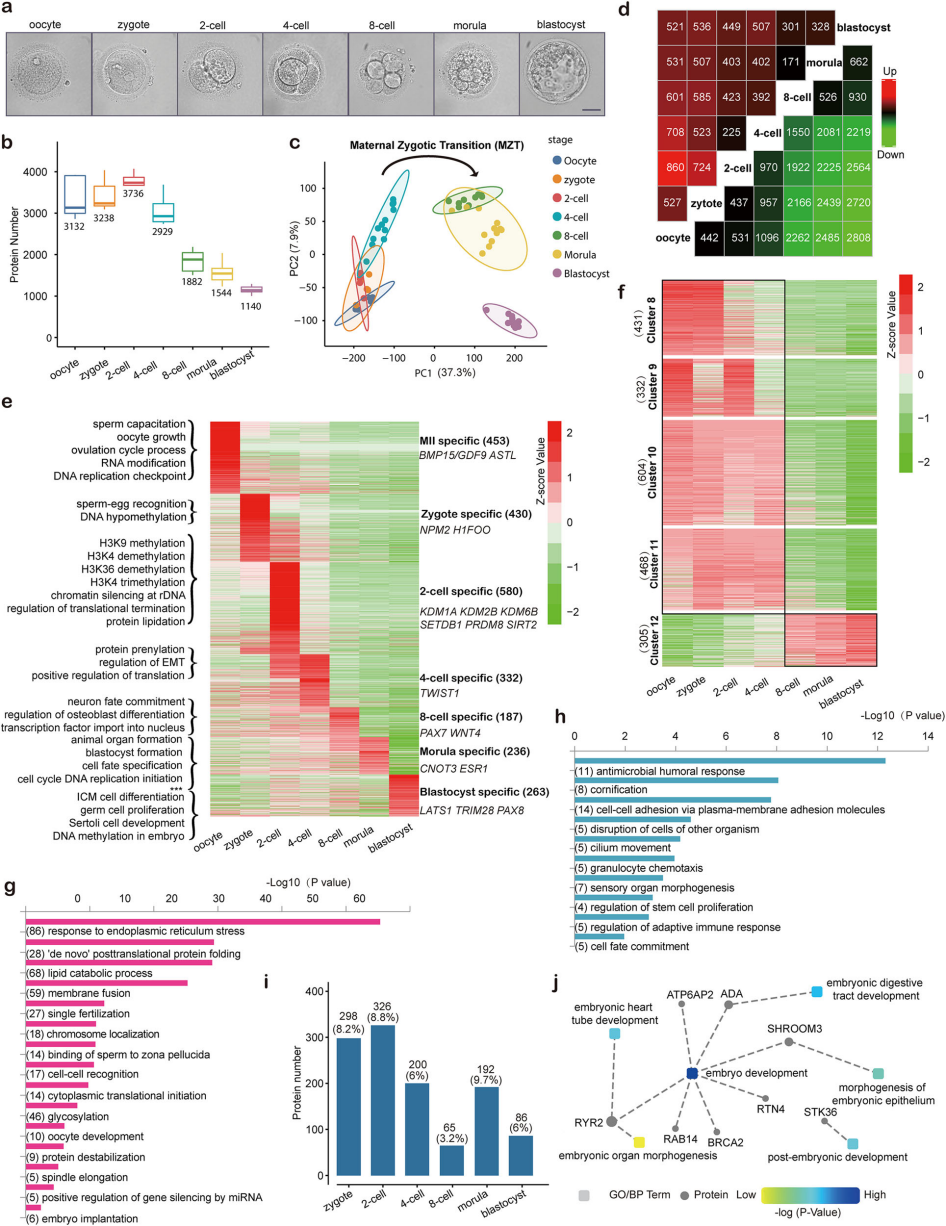
Landscape of dynamic protein expression during human pre-implantation development. **a** Representative microscopy images of a human mature (MII) oocyte (5 samples were analyzed) and early embryos at the following stages: zygote (2 pronuclear (2PN) embryo) (5 samples), 2-cell (4 samples from 2 embryos), 4-cell (13 samples from 3 embryos), 8-cell (9 samples from 1 embryo), morula (12 samples), blastocyst (10 samples). Scale bar, 50 µm. **b** Box plot showing the number of proteins detected in single oocytes or blastomeres. Median values are shown. **c** Proteomic PCA of single oocytes and blastomeres during human pre-implantation development. **d** Number of DEPs (fold change > 2 or < 0.5) at each stage compared with the other stages. **e** Heatmap of stage-specific proteins including their normalized expression levels (Z-score value), marker proteins (at right), protein numbers (at right) and specific GO-terms (at left). *** represents GO terms of ‘RNA polymerase II transcriptional pre-initiation complex assembly’ and ‘regulation of translation initiation in response to endoplasmic reticulum stress’ at the morula stage. **f** Heatmap of corresponding maternal-translated (clusters 8–11) and zygotic-translated proteins (cluster 12). Numbers of proteins are shown in brackets. **g** GO analysis of maternal-translated proteins. Numbers of proteins participated in the terms are shown in brackets. **h** GO analysis of zygotic-translated proteins. Numbers of proteins participated in the terms are shown in brackets. **i** Histogram showing the number of de novo expressed ‘prime-state genes’ at each stage. Percentages of de novo expressed ‘prime-state’ proteins to all expressed proteins are shown in brackets. **j** Identification of biological processes (BP) in embryo development based on analysis of the conserved proteins. Square nodes represent BP terms; circular nodes represent related proteins.

Differentially expressed proteins (DEPs) revealed by proteome analysis (fold change > 2) were summarized (Fig. 1d; Supplementary Table S2). Based on a fuzzy c-means algorithm, DEPs were clustered into 12 groups during early embryo development as temporal proteomic expression patterns (Supplementary Fig. S1d, e). Cluster 1 to Cluster 7 represented the unique stage-specific proteins with remarkably higher expression levels at each stage (Supplementary Fig. S1d). The predicted functions and marker proteins of each stage during pre-implantation development were listed in Fig. 1e. Collection of the other four clusters (8–11) (Supplementary Fig. S1e) was made up of thousands of maternal-translated proteins (Fig. 1f, g) including the maternal functional module subcortical maternal complex (SCMC)^8,9^ and demethylase TET3^10^. The last cluster consisted of 305 proteins (Supplementary Fig. S1e) zygotically translated after the 8-cell stage, including regulators of ‘stem cell proliferation’, ‘cell fate commitment’, and ‘organ morphogenesis’ (Fig. 1f, h). Tens of proteins were involved, including the transcription factor GBX1, which regulates neuroectoderm differentiation in model animals^11,12^.

To verify the reliability of our defined maternal-translated and zygotic-translated proteins, we performed immunofluorescence staining of PMGE, TXND5 and CAVN1 in human embryos (Supplementary Fig. S2). As expected, PMGE, as a maternal-translated protein, mainly located on the cell membrane and in the nucleus, was highly expressed in early embryos (Supplementary Fig. S2a–c). Another maternal-translated protein, TXND5, was also highly expressed before the 8-cell stage (Supplementary Fig. S2d–f). In addition, we also verified that the zygotic-translated protein CAVN1 was mainly located in the nucleus and highly expressed after maternal-to-zygotic transition (MZT) (Supplementary Fig. S2g–i). Thus, the three verified proteins were all expressed as predicted in our data.

As previously reported in mice^13^, the correlation coefficient between transcriptome and proteome was also generally low during human pre-implantation development (Supplementary Fig. S3a). To further depict the translational activity across human pre-implantation development, we revealed the patterns of de novo protein translation. We analyzed prime-state genes ready for translation, which showed high mRNA levels but no protein expression at a certain stage and displayed de novo translation at the next stage (Supplementary Fig. S3b–d and Table S3). Two distinct rises were observed. The first rise was from the zygote to the 2-cell stage after fertilization, while the second rise was from the 8-cell stage to the morula stage. These two waves of hundreds of proteins were successively de novo translated before MZT^14^ and after zygotic genome activation (ZGA)^15^, respectively (Fig. 1i). As a result, the former wave of de novo translation was defined as ‘maternal proteome activation’ (MPA) triggered by maternal mRNA, possibly serving as the pulse-on of MZT; and the second wave of de novo translation was defined as ‘zygotic proteome activation’ (ZPA), executed by zygotic genes to promote further development. To verify our assumption, we conducted GO analysis at Metascape (http://metascape.org/). Proteins of MPA functioned in ‘RNA polymerase II transcription termination’ to repress transcription before MZT (Supplementary Fig. S3e, f); and proteins of ZPA were activated to function on ‘translation initiation’ for further development after ZGA (Supplementary Fig. S3g, h). This translational transition from MPA to ZPA ensured the MZT process across human pre-implantation development.

Cross-species comparison of embryonic proteomes provides evolutionary clues and crucial insights into the pre-implantation regulatory network. We compared the latest proteomic data^13^ of mouse embryos with our proteomic data of human embryos. Prior to studying proteomic changes between humans and mice, we firstly reanalyzed the quantitative proteomic data of mouse embryos (Supplementary Fig. S4a, b). Cross-species comparisons were conducted among DEPs at 7 stages in humans and 6 stages in mice (Supplementary Fig. S4c). The highest correlation coefficient value of cross-species DEPs was detected between 2-cell embryos in mice and 8-cell embryos in humans. These are just the time points of MZT in mouse and human embryos, respectively. A total of 70 overlapping proteins were found to be expressed at similar levels between 2-cell mouse embryos and 8-cell human embryos. They were conserved proteins both in humans and mice to regulate the MZT process, further embryo development and organ formation (Fig. 1j), such as NLRP2/7, WDR1 and AKT3.

To the best of our knowledge, this is the first time that the proteome of human pre-implantation embryos has been surveyed at the single-cell level. In-depth research of translational activity answers the questions about stage-specific protein expression, and proteomic MZT pattern. This proteomic study of human embryos promotes further understanding and researches on functional protein networks during human pre-implantation development.

## Supplementary information

Supplementary Information

Supplementary Table S1

Supplementary Table S2

Supplementary Table S3

## Data Availability

The experimental data that support the findings of this study have been deposited in the integrated proteome resources (iProX) of ProteomeXchange with the accession code PXD024267.
