# Supplementary material for: Functional profiling of stage-specific proteome and translational transition across human pre-implantation embryo development at a single-cell resolution
Source: Cell Discov. 2023 Jan 24;9:10. doi: 10.1038/s41421-022-00491-2 (PMC9873803; doi:10.1038/s41421-022-00491-2)
Supplement: Supplementary file 1 — Supplementary Information [file 41421_2022_491_MOESM1_ESM.pdf]

Methods

Ethics statement

This study was approved by the Reproductive Medicine Ethics Committee of Peking University Third Hospital (Research License 2019SZ-086) after comprehensively evaluating its scientific merit and ethical justification. All oocytes and embryos were obtained with written informed consent signed by donors. Informed consent confirmed that the donors voluntarily donated oocytes and embryos for proteomic research on human early embryonic development with no financial payments. Research donors were recruited from the Peking University Third Hospital with strict research inclusion criteria to minimize the potential risks of donors. This study employed standard clinical procedures for oocyte collection, intracytoplasmic sperm injection (ICSI), embryo culture, and collection in IVF, as previously published[1].

Single MII oocyte collection

Oocytes were donated by three females who met their fertility needs, with at least one healthy offspring. After ultrasound-guided oocyte pickup by vaginal puncturing, cumulus oocyte complexes (COCs) were washed with MOPS and cultured in fertilization medium. The cumulus cells around the oocytes were removed using hyaluronidase treatment. Only mature MII oocytes were selected, and the zona pellucida was removed using a mouth pipette. After washing with phosphate buffered saline (PBS) three times, single metaphase II (MII) oocytes were transferred to a 1.5 mL low-binding tube.

Embryo culture and single blastomere collection

After intracytoplasmic sperm injection (ICSI), embryos were cultured in G1 medium (Vitrolife, Sweden) to obtain zygotes, 2-cell embryos, 4-cell embryos, and 8-cell embryos, and collected at appropriate times according to embryonic development. The remaining day-3 cleavage-stage embryos were transferred to G2 medium to grow morulae and blastocysts.

Each embryo was transferred to an acidic solution to remove the zona pellucida. The embryos were then washed with PBS and treated with accutase medium for single

blastomere isolation. Separated blastomeres were washed 3-5 times and then transferred into tubes one by one for MS analysis.

Single oocyte or single blastomere proteome extraction and preparation

A custom-built platform was used to perform multistep sample pretreatment for single-cell proteomic analysis. First, 200nL single oocytes or blastomere droplets were transferred from the prepared suspension to the chip droplet layer using a thick capillary probe. Second, single oocytes or blastomeres were lysed by the addition of 50 nL of 0.5% (w/v) RapiGest and incubated at 95 °C for 10 min. Next, the protein content of each cell was reduced by adding 50 nL of 30 mM tris(2-carboxyethyl)-phosphine (TCEP) and incubating at room temperature for 30 min. Then, alkylation was performed by adding 50 nL 70 mM iodoacetamide (IAA), and the droplet was incubated at room temperature for 30 min in the dark. Subsequently, 5 ng of trypsin/Lys-C mix in 50 nL droplets was added and incubated at 37 °C overnight in the dark. Digestion was terminated by adding 50 nL of 45% formic acid (FA) to a final concentration of 5% and incubating for 30 min at room temperature. The final volume of each sample droplet was 450 nL. With the aid of a self-aligning monolithic (SAM) device, the sample droplet was driven to the capillary LC column by applying pneumatic pressure to the closed pump tank. The nanowell was washed with 500nL of 0.1% formic acid (FA) droplets before being pumped into LC column. Subsequently, the LC column was flushed with 0.1% FA at 100 nL/min for 1 hour by the LC system to achieve desalting.

LC-MS analysis

Protein samples from single oocytes or blastomeres were subjected to a single LC-MS/MS experiment that was performed on a nanoElute system connected to a hybrid trapped ion mobility spectrometry-quadrupole time-of-flight mass spectrometer (timsTOF Pro, Bruker Daltonics) with a modified nano-electrospray ion source (CaptiveSpray, Bruker Daltonics). An in-house packed 25 cm long, 50 μm inner diameter analytical column with a pulled emitter tip packed with reversed-phase C18 ReprosilSaphir 1.5 μm particles (Dr. Maisch, Ammerbuch, Germany) was used for all LC separations. Peptides of blastomere or oocyte samples were separated at a

flow rate of 200 nL/min with mobile phases A (0.1% FA in water) and B (0.1% FA in 100% ACN) with a 120 min gradient (0-90 min, 2-22% B; 90-105 min, 22-37% B; 105-110 min, 37-95% B; 110-120 min, 95-95% B).

The timsTOF Pro was operated in dda-PASEF mode. The settings were as follows: The Mass Range was 100 to 1700 m/z with a 1/K0 range from 0.6 to 1.6 V·s/cm2; the Ramp time was set to 110.1 ms, and the Lock Duty Cycle was locked to 100%. Capillary Voltage was set at 1700V. The PASEF settings were as follows: The test contained a total cycle time of 1.27 s, one MS1 scan and ten PASEF MSMS scans, and the charge range was 2-5. The scheduling target intensity was 10000cts/s. The intensity threshold was 1000cts/s.

MS data searching

Tandem mass spectra of the raw data were searched by PEAKS software (version Online X). The experimental type was TIMS-DDA with CID fragmentation. The digestion enzyme was set at 'trypsin', and the allowed missed cleavage for each peptide was 2. The precursor tolerance was limited to 15 ppm, and the fragment tolerance was set as 0.05 Da. The MS/MS spectra were searched against the UniProtKB/Swiss-Prot human database with 20421 entries. N-terminal protein acetylation and methionine oxidation were selected as variable modifications. Carbamidomethylation of cysteine residues was set as a fixed modification. A 1% FDR was applied at the peptide-spectrum match (PSM) level, which resulted in a peptide score threshold of 20. Protein was inferenced from the list of identified peptides with parsimony algorithm. Protein score (-10*log P) was calculated by summarizing supporting peptide scores with weight factors from 0 to 1. The confidence of a single hit protein with a score of 20 is equivalent to the confidence of the peptide with a score of 20, which is roughly 1% of the FDR. Therefore, a protein score of (−10*logP) ≥ 20 containing at least one unique peptide was considered for confident protein identification. The identified proteins were quantified by the quantitation module in PEAKS software to evaluate the differences in seven crucial embryonic stages. The Match Between Runs (MBR) algorithm, with a mass tolerance of 20 ppm and retention time windows of 1.0 min. The CCS error tolerance of 0.05

$1/K_0$, was used to increase peptide or protein identification in samples with low cell numbers.

Bioinformatics analysis of MS data

The multi-omics data analysis tool OmicsBean (http://www.omicsbean.cn) was used to analyze the DEPs in the seven developmental stages. In this study, we used locally weighted polynomial regression64 (lowess in R65) to compute the local polynomial fit for the protein number and protein detection rate in each stage.

Based on the data distribution, the proteomics raw data were filled by a double boundary Bayes (DBB) impute[2,3] with the minimum, Bayesian, and median methods. The fitted distribution (cure) for each stage showed a similar trend. Below 0.5, the detection rate was negatively correlated with protein number. Above 0.5, the detection rate was positively correlated with protein number. It was used to set two boundary thresholds, at 0.15 and 0.5. When the protein detection rate was lower than 0.15, the detected value was probably due to technical error. No imputation was performed on these proteins. When the protein detection rate was above 0.5, the missing value was probably due to the detection accuracy limitation of LC-MS. In this case, the missing value is replaced with the median value. When the protein detection rate is between 0.15 and 0.5, the protein expression is unstable or the lifetime of the protein is too short; therefore, the detection of the protein is unstable.

In this case, we first calculated the missing probability of a protein using Bayes' theory(*).

missp=PA*(PBA/((PBA*PA)+(0.05*(1-PA)))) *

where PBA is the group missing rate(PBA), and PA is the total missing rate(PA) of each protein.

We then determined the predicted imputation number (IN) for each protein in each group.

IN =min (Mj/2,(1-missp) * Mi) *

where Mi= # undetected sample number of a protein in group i and

Mj= # detected sample number of protein in group i.

A random method was used to determine the imputed samples. The imputation value (IV) was defined as IV = min (Me)*.

where Me=# minimum detecting value of the protein in group i

The data was then normalized to eliminate the dimensional influence between indicators allowing experimental data from different batches to be compared and analyzed. The imputed data was normalized column-wise using the SumNorm and LogNorm algorithms with the R software. Column values of the input data are converted to a constant sum using the flowing SumNorm formula, where x is the column value.

```
SumNorm<- function(x){1000*x/sum(x, na.rm=T);}
```

Then, normalizing the obtained data using the LogNorm formula for tolerance to 0 and a negative value, where min is the input minimum value.

```
LogNorm<-function(x, min.val){log10((x + sqrt(x^2 + min.val^2))/2)}
```

Software for Analysis

PCA (mumav1.4 package, https://www.rdocumentation.org/packages/muma) and t-SNE (Rtsnev1.4 package, https://github.com/lmweber/Rtsne-example) plotting were used to show proteome relationships between single cells.

fastclusterv.1.1 (https://www.rdocumentation.org/packages/fastcluster/versions/1.1.25/) was used to perform clustering analysis, and Euclidean distance was used to calculate the distance between samples.

Mfuzz (https://www.bioconductor.org/packages/release/bioc/html/Mfuzz.html) was used to perform clustering of gene expression time-series data. The R package pheatmap (https://cran.r-project.org/web/packages/pheatmap/index.html) was employed to plot a heatmap for data visualization.

R package Genefilter was used to calculate the fold-change values of the proteins. A fold change of 2 and a p-value of 0.05 were used to filter DEPs.

Weighted gene co-expression network analysis (WGCNA) was used to detect co-expressed modules and hub genes in the differential stage.

A multi-omics data analysis tool, OmicsBean (http://www.omicsbean.cn), which integrates GO enrichment and protein-protein interaction (PPI) analysis, was used to analyze the function of stage-specific protein expression.

GO analysis of DEPs was performed at level 5 and a P-value < 0.001 on OmicsBean. Enrichment analysis is used to test whether a GO term is statistically enriched for a given set of genes, and the hyper-geometric test is the most common statistical method of enrichment analysis.
Where N is the number of all genes of the specific organism annotated in GO (Background Genes), n is the number of query genes annotated to the GO term, M is the number of all genes annotated to certain GO terms(Pop Hit),and m is the number of query genes annotated to certain GO terms(count).

$$P = 1 - \sum_{i=0}^{m-1} \frac{\binom{M}{i}\binom{N-M}{n-i}}{\binom{N}{n}}$$

Immunofluorescence and confocal microscopy

Human oocytes and pre-implantation embryos were fixed with 4% paraformaldehyde in PBS for 1 h and permeabilized with 0.3% Triton X-100 in PBS for 30 min. The cells were then blocked in 1% bovine serum albumin (BSA) for 1 h at room temperature and sequentially incubated for 1 h at room temperature or overnight at 4 °C with the following primary antibodies: rabbit polyclonal anti-PTRF/CAVN1 (ab48824, Abcam, 1:100); rabbit polyclonal anti-PMGE/BPGM (ab97494, Abcam, 1:100); and rabbit polyclonal anti-TXND5 (GTX55826, GeneTex, 1:100). Next, thecells were washed three times in PBS containing 0.1% Tween 20 and 0.01% Triton X-100and labeled with goat anti-rabbit Alexa Fluor 488-conjugated secondary antibody (R37116, Invitrogen, 1:200) for 1 h at room temperature. Finally, the samples were counterstained with Hoechst 33342 (10 μg/ml) for 15 min and observed under a confocal laser scanning microscope (Carl Zeiss LSM880, Jena, Germany).

Reference:

1. Dang, Y. *et al.* Tracing the expression of circular RNAs in human pre-implantation embryos. *Genome biology* **17,** 130 (2016).

2. Webb-Robertson, B. J. M. *et al.* Review, evaluation, and discussion of the challenges of missing value imputation for mass spectrometry-based label-free global proteomics. *Journal of Proteome Research* **14,** 1993–2001 (2015).

3. Gao, Q. *et al.* Integrated Proteogenomic Characterization of HBV-Related Hepatocellular Carcinoma. *Cell* **179,** 561–577.e22 (2019).

Supplementary Figure 1

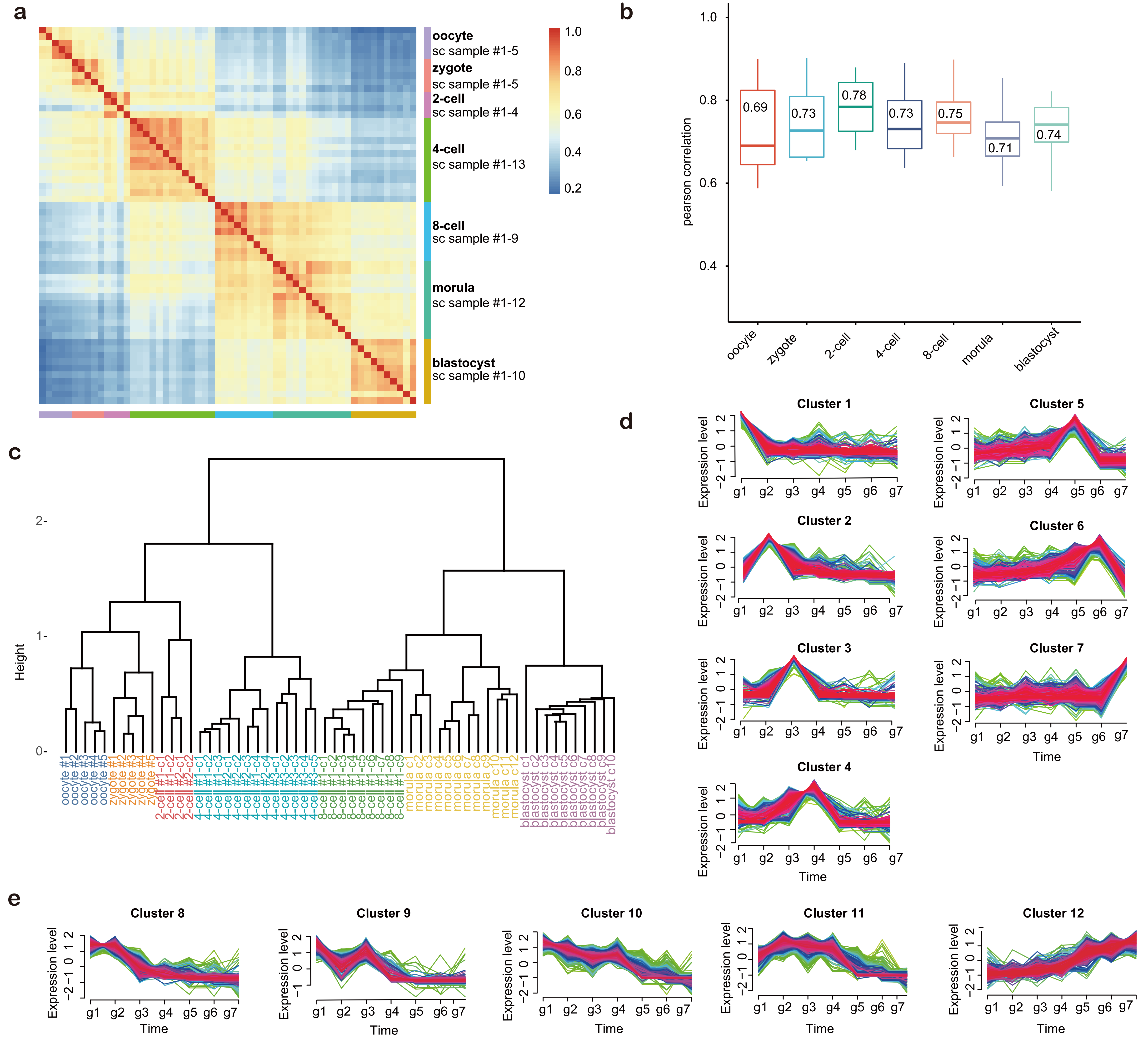

Figure S1: (a) Pearson correlation coefficient heat map of single-cell proteomes during human pre-implantation development. (b) Box plot showing correlation coefficients within each stage. (c) Unsupervised hierarchical clustering of single-cell proteomes during human pre-implantation development. (d) Expression patterns of stage-specific proteins clustered by fuzzy c-means algorithm. Groups 1-7 (g1-7) represent stage-specific proteins from oocyte, zygote, 2-cell, 4-cell, 8-cell, morula and blastocyst, respectively. (e) Expression patterns of the other 5 clusters classified by fuzzy c-means algorithm.

Supplementary Figure 2

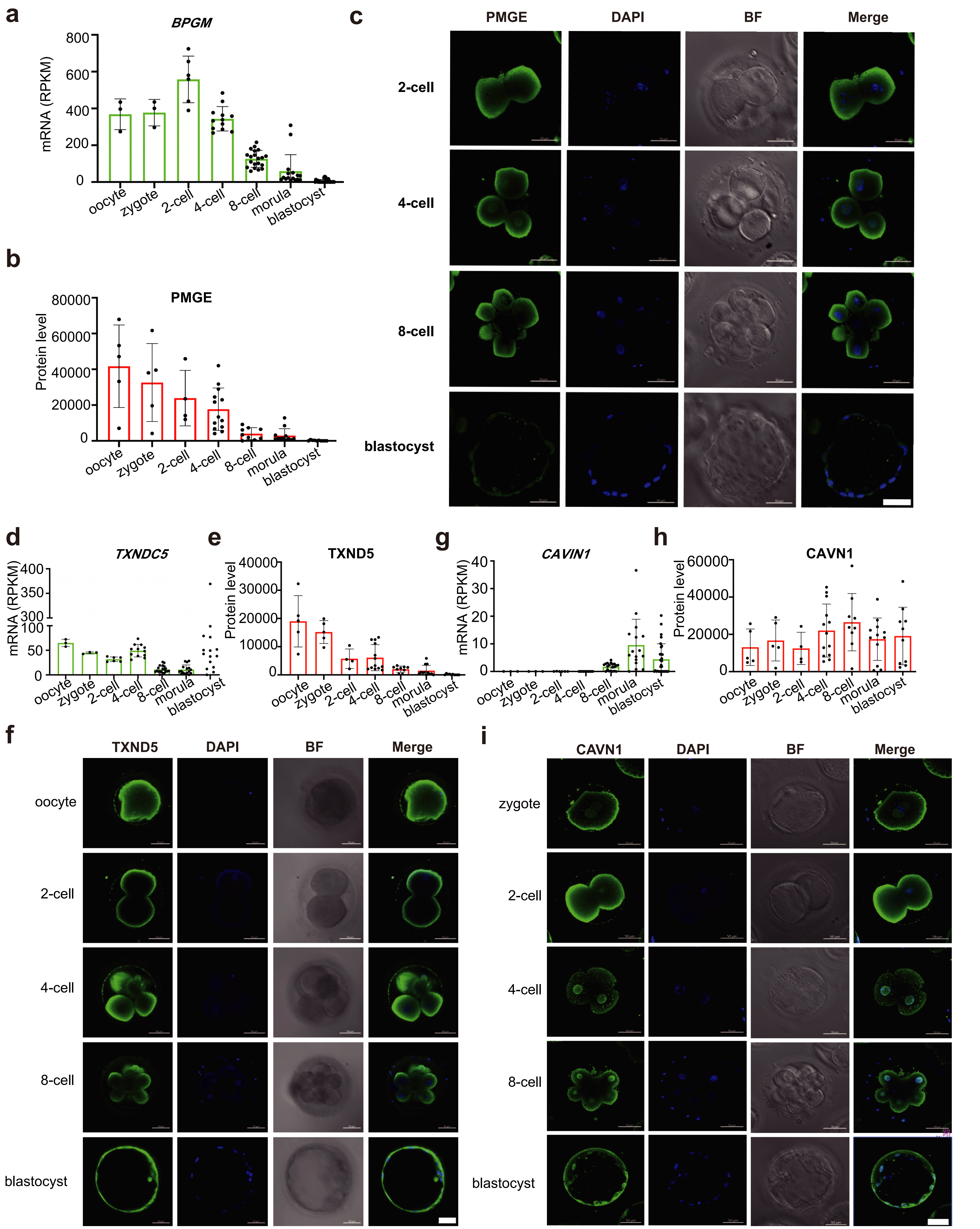

Figure S2: Immunostaining verification of three proteins. (a, d, g) The expression pattern of BPGM, TXNDC5, and CAVIN1 mRNA at different stages of human pre-implantation embryos (from oocyte to blastocyst). The data is presented as RPKM. (b, e, h) Protein expression levels of PMGE (encoded by BPGM), TXND5 (encoded by TXNDC5), and CAVN1 (encoded by CAVIN1) at different stages of human pre-implantation embryos (from oocyte to blastocyst). (c) Immunofluorescence staining of PMGE (green) in the 2-cell, 4-cell, 8-cell, and blastocyst stages.(f) Immunofluorescence staining of TXND5 (green) in oocytes, 2-cell, 4-cell, 8-cell, and blastocysts. (i) Immunofluorescence staining of CAVN1 (green) in zygotes, 2-cell, 4-cell, 8-cell, and blastocysts. DAPI (blue) indicates the nuclei. BF, bright field. Scale bar: 50 μm.

Supplementary Figure 3

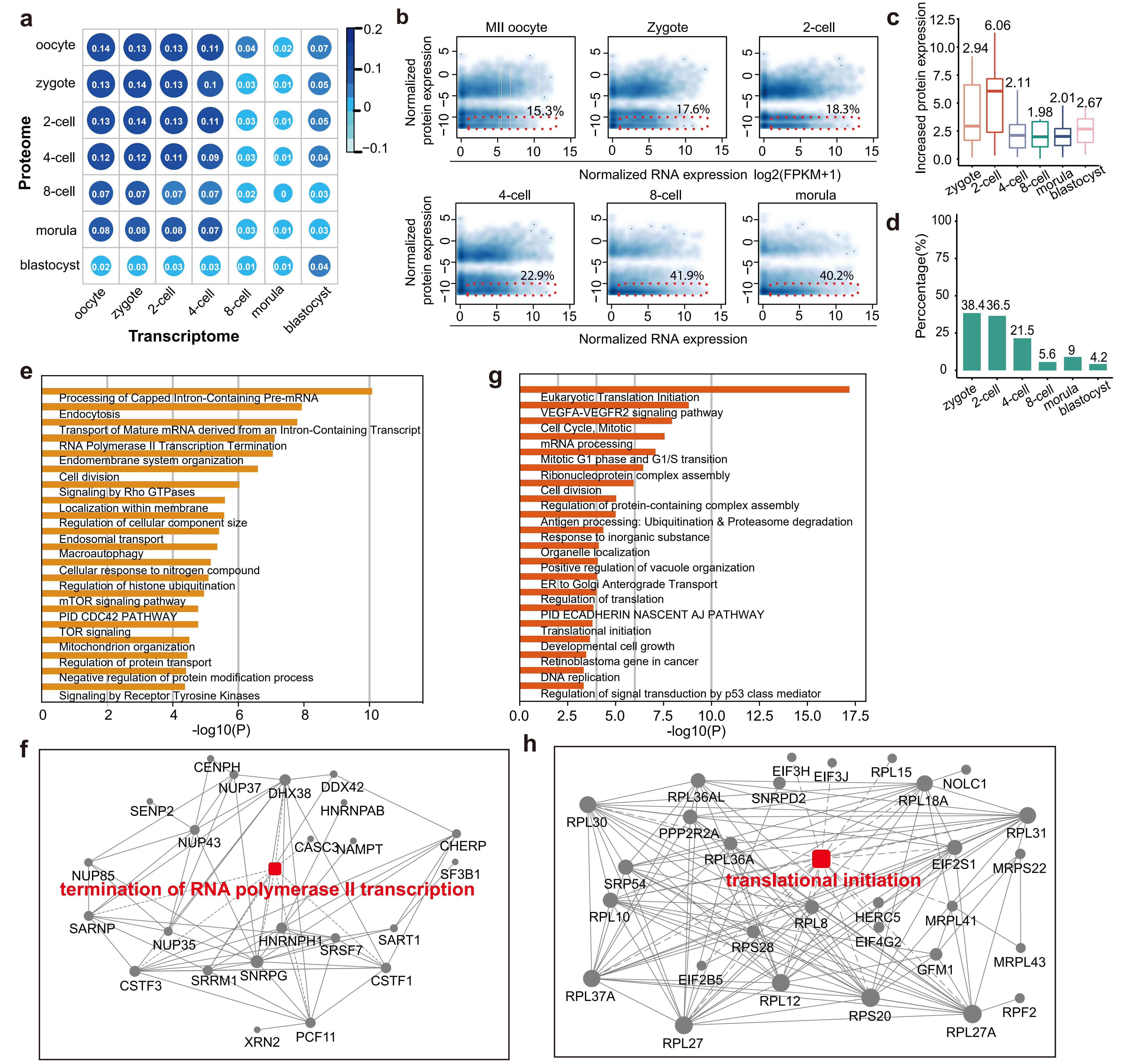

Figure S3: (a) Pearson correlation coefficients between the transcriptome and proteome across stages during human pre-implantation development. (b) Scatter plot showing gene expression and protein levels. Dashed red rectangles represent non-expressed proteins (normalized value <-10) with RNA expressed (FPKM>1) at each stage, defined as ‘prime-state genes’. (c) Box plot showing de novo expression levels of prime-state proteins compared to the former stage. For example, ‘zygote’ represents the expression level of the zygote subtracted from that of the oocyte. Median values are shown. (d) Histogram showing the percentages of de novo expressed ‘prime-state’ proteins to all prime-state genes of the former stage. (e) GO analysis of MPA proteins at 2-cell stage. (f) PPI analysis of ‘termination of RNA polymerase II transcription’ at MPA. (g) GO analysis of ZPA proteins at morula stage (h) PPI analysis of ‘translational initation’ at ZPA.

Supplementary Figure 4

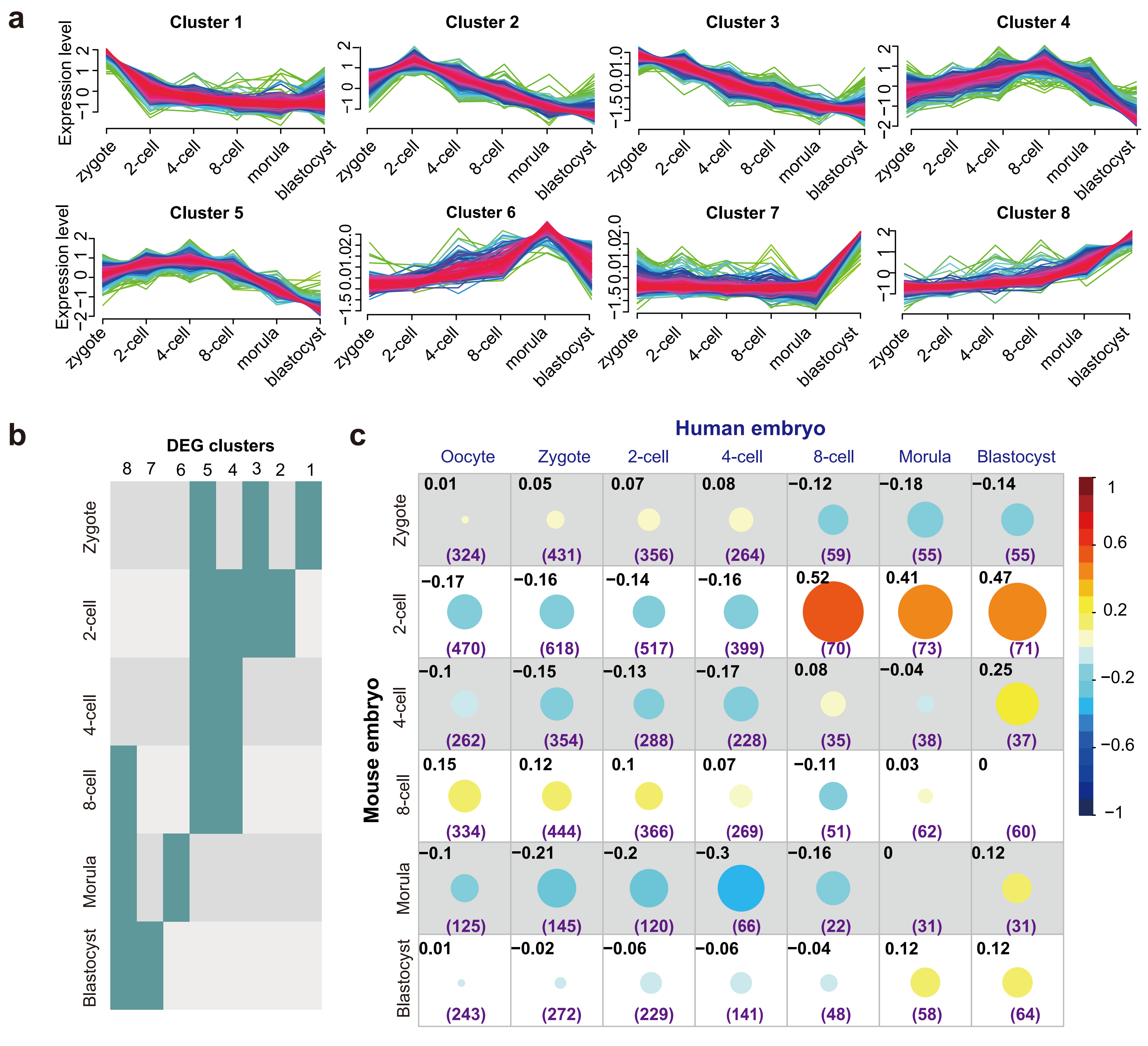

Figure S4: (a) Expression patterns of differentially encoded proteins in mouse embryos subjected to eight clusters based on the fuzzy c-means algorithm. (b) Number of DEPs at six stages as the sum of DEPs according to eight clusters gathered by the fuzzy c-means algorithm. (c) Pearson correlation coefficients (at top left) and number of overlapping proteins (at bottom) between human and mouse during pre-implantation development.
